# Effects of Spirulina Enrichment on Bread Quality: Color, Texture, Volatile, and Sensory Properties

**DOI:** 10.1002/fsn3.72345

**Published:** 2026-09-15

**Authors:** Francesca Masino, Emanuela Lo Faro, Alessandra Riggio, Marco Gammaitoni, Giulia Santunione, Fabio Licciardello, Andrea Antonelli, Giuseppe Montevecchi

**Affiliations:** ^1^ Department of Life Science (Agro‐Food Science Area) University of Modena and Reggio Emilia Reggio Emilia Italy; ^2^ BIOGEST–SITEIA Interdepartmental Centre University of Modena and Reggio Emilia Reggio Emilia Italy

**Keywords:** bakery products, color, flavor, *Limnospira platensis*, sensory analysis

## Abstract

Spirulina (*Limnospira platensis*) is an ingredient of nutritional interest that may affect the quality traits of bakery products. This study aimed to evaluate the effects of low‐level Spirulina enrichment on the color, texture, volatile profile, and sensory properties of bread made with Italian type “1” wheat flour. Bread samples were prepared with 0% (control), 1%, and 2% (w/w) Spirulina and analyzed by instrumental color measurement, texture profile analysis, HS‐SPME‐GC‐EI‐MS, and trained‐panel sensory descriptive analysis. Spirulina‐enriched samples showed darker crust and crumb and clear changes in chromatic coordinates. Texture profile analysis indicated limited changes in the instrumental texture profile; specifically, the sample with 1% w/w Spirulina showed significantly lower hardness than the control, whereas the other measured texture parameters did not differ significantly among samples. Volatile analysis revealed differences in several compounds, including carotenoid‐derived volatiles such as β‐ionone, β‐cyclocitral, dihydroactinidiolide, and safranal in enriched samples. Sensory analysis highlighted stronger bitter and marine‐related notes in Spirulina‐enriched breads. Spirulina enrichment modified the chromatic, volatile, and sensory properties of bread, while producing less considerable changes in the instrumental texture parameters measured. The results indicate that Spirulina may be incorporated at low levels in bread, although sensory attributes such as bitterness and marine notes may represent acceptance barriers and should be further evaluated through dedicated consumer testing.

## Graphical abstract

This study explored how the addition of 1%–2% Spirulina powder affects bread quality, focusing on color, texture, volatile compounds, and sensory characteristics. Spirulina mainly influenced bread color and volatile profile, while texture remained largely unchanged. The enriched samples also showed more pronounced bitter and marine notes. Overall, the results indicate that Spirulina can be incorporated into bread at low levels, although further formulation improvements may help reduce its impact on sensory characteristics.

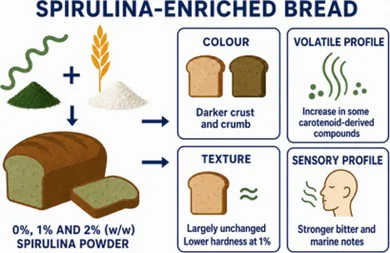

## 1 Introduction

The search for sustainable protein sources to enrich staple foods has intensified in recent years, as conventional animal farming presents high environmental costs. Alternatives such as microalgae, cyanobacteria (Montevecchi et al. 2022), and insects (Montevecchi et al. 2021) have been investigated for their ability to improve protein quality in common foods without compromising processing properties. Incorporating modest amounts of these ingredients (typically 1%–4% of flour weight) has been shown to enhance essential amino acid content while maintaining dough functionality and not adversely affecting its rheological properties.

In recent years, there has been a growing focus on exploring the nutritional benefits and characteristics of green microalgae (Santunione et al. 2024) and cyanobacteria, commonly known as blue‐green microalgae (Udayan et al. 2017). Among these novel ingredients, *Limnospira platensis*, commonly known as Spirulina, has received particular attention. Spirulina is widely recognized for its high protein content (up to 70% dry weight) with a complete amino acid profile (Rodríguez De Marco et al. 2014; Lafarga et al. 2020), as well as its abundance of minerals (Moreira et al. 2013) and bioactive compounds (Donaldson 2000), while B12 is in its pseudo‐vitamin B12 form that is not bioavailable for humans (Santunione and Montevecchi 2025). Its long history of safe use in food and supplements, along with GRAS status in the United States and approval in the European Union (Novel Food Catalogue), has further encouraged its application in new product development (European Parliament and Council of the European Union 1997, 2015).

Despite these advantages, Spirulina also presents limitations for food fortification. Its blue‐green pigments (chlorophylls and phycobiliproteins) alter the appearance of products, often producing an atypical green hue (Leema et al. 2010; Shang et al. 2023). Moreover, its characteristic earthy, seaweed‐like flavor can reduce consumer acceptance when incorporated at levels sufficient to deliver nutritional benefits. Previous studies have reported that sensory properties—particularly color and flavor—are among the main barriers to the widespread use of Spirulina in staple foods such as bread, where consumers strongly associate traditional appearance and taste with product quality.

Bread provides a particularly relevant case study, as it is one of the most widely consumed staple foods, but consumer expectations regarding its sensory profile remain highly conservative. Previous work has shown that Spirulina can successfully enhance the nutritional profile of wheat‐based doughs by increasing protein and essential amino acid content without causing drastic rheological alterations when used at low inclusion levels (≤ 2% w/w) (Montevecchi et al. 2022). However, these benefits came alongside reductions in gluten content and visible changes in dough characteristics, suggesting that further consequences might emerge at the sensory stage (Montevecchi et al. 2022). Since even small deviations in bread's appearance, smell, or flavor can affect consumer acceptance, a systematic evaluation of Spirulina's impact must extend beyond nutritional and rheological properties to include sensory perception and overall product quality.

To build on previous work on Spirulina‐enriched wheat systems and to address the sensory dimension more directly, the present study evaluated the effects of low‐level Spirulina enrichment on bread color, instrumental texture, volatile profile, and descriptive sensory attributes. Rather than proposing a new processing strategy, this work aimed to provide an integrated product‐level characterization of Spirulina‐enriched bread, with particular attention to quality traits that may influence its sensory perception.

## 2 Materials and Methods

### 2.1 Sampling of Raw Materials and Preparation of the Flour Mixtures

Wheat flour (Italian type “1”; *W* = 300; Industria Molitoria Denti Srl, Borzano di Albinea, Reggio Emilia, Italy) had moisture content of 14.5 g max/100 g, ash content of 0.80 g max/100 g dry weight, and protein content of 12 g min/100 g dry weight. Spirulina (*Limnospira platensis*, formerly *Arthrospira platensis*) flakes were composed of 55.4 g/100 g dry weight protein, 1.1 g/100 g lipids, and 11.6 g/100 g carbohydrates; additionally, it contained 1.3 g/100 g phosphorus, 544 mg/100 g magnesium, 63 mg/100 g iron, 0.15 mg/100 g vitamin B1, 10 mg/100 g vitamin B3, and 16.9 mg/100 g vitamin E.

Additional ingredients, including water (minimally mineralized, with a total dissolved solids of 48 mg/L), table salt, and baker's yeast ( *Saccharomyces cerevisiae* ), were purchased from local markets.

Before incorporation into the flour, Spirulina flakes were finely ground using an immersion blender (Pimmy metal, Ariete, Florence, Italy) and sieved through a high‐precision sieve with 500 μm opening size (35 mesh) (Certified test sieves, Giuliani Tecnologie, Turin, Italy). Flour mixtures were prepared using the geometric dilution method, ensuring thorough homogeneity with kitchen utensils and an electric mixer.

Two flour mixtures were created: (1) with 10 g of Spirulina powder (SF10; 1% w/w) and (2) with 20 g of Spirulina powder (SF20; 2% w/w), both added to wheat flour, resulting in a total of 1 kg of mixture. The control sample consisted solely of wheat flour.

### 2.2 Dough Preparation, Leavening, and Sample Baking

Bread doughs were prepared according to a protocol adapted from Montevecchi et al. (2022), with the full formulation reported here for reproducibility. Each dough batch was prepared using 250 g of total flour mixture, 100 mL of minimally mineralized water, 50 mL of NaCl solution (10 g/100 g), and 0.5 g of baker's yeast ( *Saccharomyces cerevisiae* ). The control dough contained 250 g of wheat flour, whereas in the Spirulina‐enriched doughs, wheat flour was partially replaced with Spirulina powder at 1% w/w (2.5 g Spirulina + 247.5 g flour) or 2% w/w (5.0 g Spirulina + 245.0 g flour) on a total flour basis. Doughs were mixed until homogeneous and left to rise for 24 h at 25°C in sealed 1.25‐L plastic containers.

After leavening, 250 g of dough was transferred into ceramic ramekins (upper side lengths = 14.0 × 10.0 cm; lower side lengths = 10.5 × 4.5 cm; height = 7.0 cm; nominal volume = 420 mL) and baked at 220°C for 30 min (ATBO 30N4T oven, Atlantic, Milan, Italy). The same ramekin type was used for all samples. The reported data are based on three biological replicates, each corresponding to an independent dough and bread batch prepared under identical experimental conditions. Each batch was subsequently analyzed through multiple measurements, depending on the parameter assessed.

### 2.3 Color Measurement

Color measurement of flour, dough, and baked samples was performed using a colorimeter (CR‐400, Konica Minolta B.S. Italia S.p.A., Milan, Italy) set to the D65 illuminant (CIE standard) as described by Musetti et al. (2015). The colorimeter's CIE L*, a*, and b* color space coordinates were provided. The instrument was calibrated using its reference tile. Five measurements were taken at different points for each sample to obtain colorimetric coordinates, and color indices for both the crust and crumb of the bread samples were derived.

### 2.4 Texture Profile Analysis (TPA) of Baked Bread Samples

TPA was carried out using a dynamometer (Z1.0 UTM, ZwickRoell Srl, Ulm, Germany) equipped with a 1 kN load cell and an aluminum cylindrical probe (4 cm height, 2 cm diameter). The 25 mm thick bread slices (crumb) underwent a bicyclic compression test up to 25% deformation of the original height of bread at a crosshead speed of 5 mm/s.

Hardness (*N*) was defined as the maximum force (*F*_max_) recorded during the first compression cycle (AACC 2000). In the present test, the first compression was performed up to 25% deformation of the original crumb height.

In addition, springiness, cohesiveness, gumminess, and chewiness were also evaluated. Springiness (dimensionless) can be measured as the ratio between the distances along the *x*‐axis (distance axis) from the point of zero force to the point of maximum force on the second compression curve and the first compression curve, respectively. This reflects how far the sample recovers in height between compressions. Cohesiveness (dimensionless) is a measure of the strength of the internal bonds making up the body of the product and it was defined as the ratio of the positive force area during the second compression portion to that during the first compression (Area 2/Area 1), excluding the areas under the decompression portion in each cycle. Gumminess (*N*) is the product of hardness (Fmax) × cohesiveness (Area 2/Area 1). Chewiness (*N*) is the energy required to masticate a solid product to a state ready for swallowing and it can be defined as the product of gumminess × springiness, which is equivalent to hardness × cohesiveness × springiness.

### 2.5 Extraction and Determination of Volatile Compounds Through HS‐SPME‐GC‐EI‐MS

Volatile compounds released from the bread samples were analyzed by solid‐phase microextraction (SPME) coupled with gas chromatography–mass spectrometry (GC–MS). Extraction was carried out under standardized conditions for all samples using a sealed‐beaker headspace configuration, rather than a conventional vial, in order to accommodate relatively large bread portions containing both crust and crumb while minimizing additional sample disruption before analysis. After baking, 90.00 g aliquots of each bread sample were obtained by dividing the bread into six pieces (15.00 ± 0.01 g each), each including both crust and crumb. These fragments were placed in a 600 mL glass beaker (internal diameter = 87 mm; height = 120 mm), which was sealed with aluminum foil and parafilm. Although vial‐based HS‐SPME is more commonly used, HS‐SPME is a flexible technique whose extraction efficiency depends on matrix characteristics, headspace‐to‐sample ratio, temperature, equilibration time, and extraction time (Raffo et al. 2015; Lancioni et al. 2022). Therefore, all these parameters, together with vessel geometry and sealing conditions, were kept constant across samples to ensure repeatability. No internal standard was used.

Each sample was allowed to rest at 40°C for 15 min to allow headspace equilibrium and stabilize volatile release. Subsequently, SPME extraction was performed using a 2‐cm StableFlex fiber (Sigma‐Aldrich, Merck, Milan, Italy) coated with a stationary phase of 50–30 μm DVB‐Carboxen‐PDMS. The extraction was conducted at 40°C for 20 min in the headspace of the beaker (the distance between the beaker's edge and the holder end was 2.2 cm), through a rubber septum glued onto the cover.

The GC–MS analysis was carried out using GC‐MSD equipment (7890A/5975C, Agilent Technologies Inc., Santa Clara, CA, USA). The fiber was inserted into the split/splitless injection port, operating in splitless mode (splitless time 0.5 min), for 4 min at 250°C. The analysis was conducted using a Stabilwax‐DA capillary column (0.25 mm i.d. × 30 m long × 0.25 μm df, Restek, Milan, Italy) with ultrapure helium as the carrier gas at a flow rate of 1.0 mL/min. Both injection port and transfer line temperatures were set at 250°C. The initial oven temperature was set at 40°C and then increased at a rate of 23.75°C/min up to 230°C, where it was held for 20 min (total runtime 28 min).

The ionization energy was set at 70 eV, and the mass range scanned ranged from 33 to 350 *m*/*z*, covering the entire range of molecular weight of foreseen volatiles. Volatile compound identification was achieved by injection of pure chemical standards. Where there was no availability of pure standards, volatile compounds were identified by comparing the mass spectrum with entries in the spectra libraries (Wiley 7th Edition, NIST‐14, as well as home‐made libraries), using specific deconvolution software (Agilent). When possible, the presence of volatile compounds was also verified by referencing literature articles focused on similar compounds.

Blank (no sample) and quality control standards were carried out every three injections to assess contamination and method stability. The samples were injected in random order, and three replicates were performed for each sample. Volatile compounds were expressed as relative percentages normalized to a total of 100%; therefore, the results provide a semi‐quantitative description of the relative headspace composition rather than absolute concentrations of individual volatile compounds.

### 2.6 Consumer Survey on Attitudes Toward Algae‐Enriched Bakery Products

An online survey was conducted to investigate consumer attitudes toward algae‐enriched bakery products, focusing on the main drivers and barriers to consumption as well as purchase‐related perceptions. The survey was intended to provide contextual information and did not involve sensory evaluation of the bread samples produced in the present study. Therefore, its results should not be interpreted as a direct assessment of consumer liking or acceptance of the experimental breads.

The questionnaire was structured into three sections: (i) sociodemographic information, (ii) frequency and habits of consuming sweet and savory baked goods, and (iii) knowledge, perceptions, sensory expectations, and purchase intentions related to algae‐containing products.

A total of 225 consumers participated, including respondents from different genders, age groups, and educational groups. The questionnaire was anonymous, and sociodemographic data included gender, age group, educational level, occupation, province and region of residence, and household size.

Consumer perceptions were assessed using a set of statements rated on a 10‐point Likert‐type agreement scale (1 = totally disagree; 10 = totally agree) to identify key factors driving acceptance or rejection of foods containing microalgae. In addition, participants were asked to indicate their willingness to purchase such products and the premium price they would be willing to pay compared with equivalent products without microalgae.

### 2.7 Sensory Analysis

#### 2.7.1 Sensory Vocabulary and Judges' Training

An 11‐member panel comprising students, PhD students, and staff from the Department of Life Sciences of the University of Modena and Reggio Emilia participated in the sensory evaluation. The panel included both men and women aged 20–47 years. These were selected based on their interest and availability, and they underwent appropriate training according to ISO 3972 (2011) and ISO 8586 (2012) standard methods.

The sensory descriptors were defined after the laboratory team tasted samples prepared with and without Spirulina, through an iterative process of evaluation and continuous discussion. The relevance of the attributes was also confirmed through a preliminary session conducted by the panel of evaluators. Finally, the panel defined each sensory attribute in its meaning and agreed on the evaluation method to avoid errors arising from different interpretations and procedures. Table 1 shows the list of ten sensory attributes evaluated by judges: bread smell (BS) and bread flavor (BF); marine smell (MarS) and marine flavor (MarF); moist (MT); hardness (HS); gumminess (GS); cohesiveness (CS); fibrousness (FR); bitterness (BT).

**TABLE 1:** Sensory vocabulary and definitions.

Attributes	Definition	Standards and scale
Bread smell (BS) Bread flavor (BF)	Smell and flavor intensity of freshly baked bread	Stale or old bread (1) – fresh bread (10)
Marine smell (MarS) Marine flavor (MarF)	Vegetable/fishy smelling and flavor typical of seawater, seaweed, seafood, (effect due to presence of Spirulina)	Wheat flour (1) – Spirulina powder (10)
Bitterness (BT)	Intensity of the bitter taste sensation perceived in the mouth	Water (1) – aqueous suspension of Spirulina (2%, w/v) containing caffeine (0.1 g/L) (10)
Moist (MT)	Perception of humidity in the crumb by contact with hands or lips	Dry bread (1) – dough (10)
Hardness (HS)	Resistance of product opposed to chewing during the first 2–3 acts of chewing	A slice of fresh bread (1) – stale or old bread (10)
Gumminess (GS)	Texture sensation of the bread crumb when forming a single compact mass (not very crumbly) when touching and chewing	Stale or old bread (1) – a slice of fresh bread (10)
Cohesiveness (CS)	Texture sensation associated to deformation/crushing mode of crumb when it is breaking in the mouth	Stale or old bread (1) – a slice of fresh bread (10)
Fibrousness (FR)	Texture sensation associated with the presence of particles of different consistency and shape in the mass during chewing (dietary fiber in flour and algae)	A slice of white wheat bread (1) – a slice of whole wheat bread (10)

The judges were trained to recognize sensory attributes such as smell, flavor, taste, and texture using both food products (e.g., fresh and stale bread) and individual ingredients (e.g., Spirulina powder). This training phase included five sessions aimed at familiarizing each judge with the sensory characteristics of bread enriched with algae. Finally, the judges were trained in the use of a sensory scale to measure attribute intensity, using reference standards (Table 1).

#### 2.7.2 Sensory Descriptive Analysis

The sensory evaluation consisted of a descriptive analysis of bread samples performed by a trained panel and was not intended to assess consumer preferences or population‐level liking. The evaluation was conducted individually to prevent exchange of opinions among judges and minimize external influences. Each trained judge recorded the intensity of selected attributes on a structured 10‐cm scale with locked endpoints.

The evaluation card was divided into three parts corresponding to perceptions through different senses: visual, olfactory, and gustatory. Judges indicated the intensity of each parameter analyzed.

Smell referred to the direct perception evaluated with the nose, while flavor indicated the retronasal perception after chewing.

The sensory evaluation was conducted under blind conditions, with panelists assessing samples in individual booths under standardized green lighting to minimize visual bias related to the characteristic coloration of Spirulina‐enriched samples. Green lighting was used to reduce immediate visual discrimination among samples during descriptive evaluation; however, it does not reproduce normal viewing conditions and may therefore limit the ecological validity of the visual assessment.

The sensory evaluation of the samples was conducted in two separate sessions, each lasting approximately 1 h. The bread samples were labeled with randomly generated numerical codes and presented on suitable plates.

### 2.8 Statistical Analysis

The data set underwent univariate analyses. Differences among samples were evaluated using one‐way analysis of variance (ANOVA); when significant effects were observed (*p* ≤ 0.05), post hoc comparisons were carried out using Tukey's multiple comparison test. For sensory descriptive data, each judge evaluated each sample only once; therefore, judge repeatability and the judge × sample interaction could not be estimated. To account for the available design, sensory attributes were further examined using an additive model including sample and judge effects, and agreement among judges was assessed by Kendall's coefficient of concordance (*W*), corrected for tied ranks. Statistical significance was set at *p* ≤ 0.05.

All statistical analyses were performed using Statistica v8.0 software (former Stat Soft Inc., now TIBCO Software Inc., Palo Alto, USA).

## 3 Results and Discussion

### 3.1 Color Analysis

**FIGURE 1:**
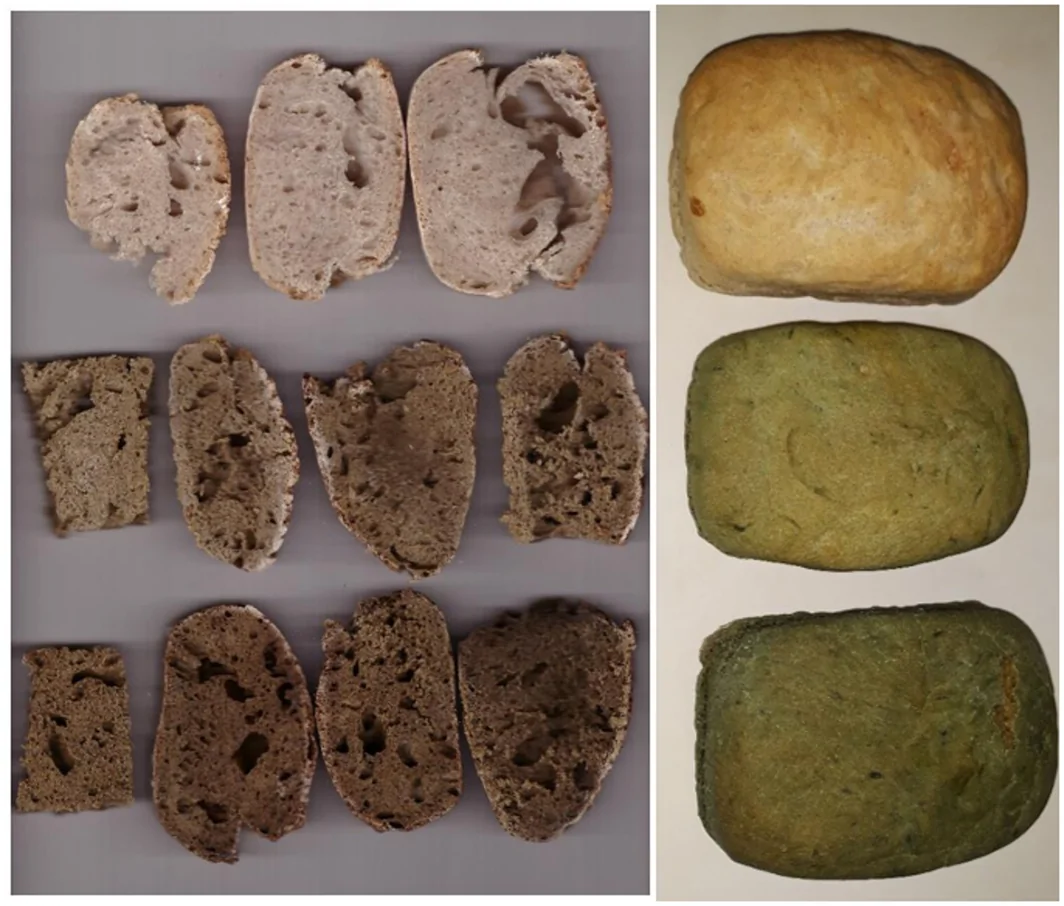
Baked products: Control (top); 1% w/w *Spirulina* (center) and 2% w/w *Spirulina* (bottom).

Table 2 shows the CIELab color coordinates of the analyzed samples. Significant differences were observed for all the parameters considered. In general, L* (lightness) and a* (redness) progressively decreased as the level of Spirulina increased in flour, dough, and baked samples, reflecting the bluish‐green color of the cyanobacterial biomass (Figure 1).

**TABLE 2:** Results of the tests for the evaluation of color. Control: Italian type “1” wheat flour (*W* = 300); SF10: Spirulina 1% w/w; SF20: Spirulina 2% w/w.

	CIELAB coordinates	Control	SF10	SF20	ANOVA (*p*)
Flour	L*	91.38 ± 0.85 a	87.01 ± 0.86 b	85.05 ± 1.37 c	2.04E−06
	a*	4.60 ± 0.05 a	3.24 ± 0.11 b	2.30 ± 0.10 c	1.12E−13
	b*	5.96 ± 0.70 c	9.36 ± 0.94 b	11.95 ± 0.99 a	6.95E−07
Dough	L*	77.55 ± 0.32 a	47.13 ± 1.14 b	37.32 ± 0.44 c	1.24E−13
	a*	6.42 ± 0.14 a	−8.61 ± 0.05 b	−8.65 ± 0.17 b	1.11E−16
	b*	14.47 ± 0.17 a	9.36 ± 0.41 b	8.26 ± 0.10 c	2.20E−10
Post‐baking crust	L*	57.67 ± 1.45 a	43.50 ± 1.64 b	34.93 ± 1.38 c	6.17E−11
	a*	13.77 ± 0.92 a	6.65 ± 0.72 b	6.06 ± 0.37 b	9.41E−10
	b*	27.61 ± 0.83 a	22.08 ± 0.76 b	14.12 ± 0.84 c	2.30E−11
Post‐baking crumb	L*	60.58 ± 4.34 a	37.07 ± 2.87 b	31.43 ± 2.02 c	1.48E−08
	a*	5.95 ± 0.51 a	4.41 ± 0.35 b	4.29 ± 0.50 b	1.38E−04
	b*	14.33 ± 1.47 c	25.64 ± 1.41 a	22.79 ± 1.64 b	1.53E−07

*Note:* Post hoc Tukey's test (*p* ≤ 0.05) was employed. Results are reported using different letters that label average values that are significantly different (a > b > c).

This effect was most evident in the dough samples. Upon Spirulina addition, the color shifted markedly toward green, as indicated by the negative a* values recorded for SF10 and SF20 (−8.61 and −8.64, respectively). Hydration of the flour–Spirulina mixture likely promoted a more uniform distribution of the cyanobacterial pigments, thereby affecting light scattering within the dough and enhancing the intensity of the green coloration.

After baking, sample color was markedly modified by heat‐induced reactions. In particular, thermal degradation of Spirulina pigments and the development of brown tones during baking may have contributed to the final color of both crust and crumb. Therefore, although Spirulina addition significantly reduced a* values compared with the control, post‐baking crumb values could remain in the positive region because the green contribution of Spirulina was masked by browning phenomena. A similar effect was also evident in the control sample, in which the red component increased markedly in the crust as a consequence of baking.

The parameter b* (yellowness) had a trend that was more irregular and difficult to interpret. In fact, the browning caused by baking increased the value of b*, while the concentration of Spirulina tended to reduce this value by moving it toward the blue region. However, the baking process caused marked color modification, with the Spirulina‐associated coloration appearing to be better retained in the crust than in the crumb (Figure 1). Differences in moisture and thermal conditions between crust and crumb may have contributed to this behavior (Ho and Redan 2021).

During fermentation with *S. cerevisiae* , the dough pH tends to slightly decrease due to the production of organic acids. The addition of Spirulina raises the initial alkalinity of the dough (Montevecchi et al. 2022), thus mitigating the final acidity. The stability of pigments (mainly phycocyanin, chlorophylls, and pheophytin) in doughs containing Spirulina during baking is affected by several factors, including temperature, dough acidity, and the presence of other ingredients.

Phycocyanin, the blue‐green pigment in Spirulina, is highly sensitive to both pH and temperature (Adjali et al. 2022). During fermentation with *S. cerevisiae* at room temperature (~25°C), its stability is low in acidic environments (pH < 6), where it degrades and loses color. While baking at temperatures above 70°C, the pigment stability decreases across all pH levels. In acidic environments, phycocyanin degrades quickly, leading to a complete loss of color, while in neutral and alkaline conditions, phycocyanin is best preserved, particularly during fermentation, but high temperatures during baking lead to significant degradation regardless of pH.

Chlorophylls are also sensitive to heat and pH. In acidic environments, chlorophylls can degrade into pheophytin, causing a color variation toward brown or olive tones. However, in neutral or slightly alkaline environments, chlorophylls are more stable and retain their green color better during baking. During baking, temperatures above 70°C accelerate the breakdown of chlorophyll into pheophytin, and at higher temperatures, pheophytin further degrades.

In the present study, the greater color modification observed in the crumb may be related to the different thermal and moisture conditions occurring in the inner part of the bread. The retained moisture, slower heating, and possible local pH differences could influence the stability of chlorophylls and phycocyanin and may contribute to the observed color changes. Conversely, rapid dehydration of the crust and its different thermal history could contribute to the different color response observed at the bread surface. Since pigment concentration, local pH, moisture evolution, and pigment degradation kinetics were not directly measured during baking, these mechanisms should be regarded as possible explanations for the observed color differences.

**FIGURE 2:**
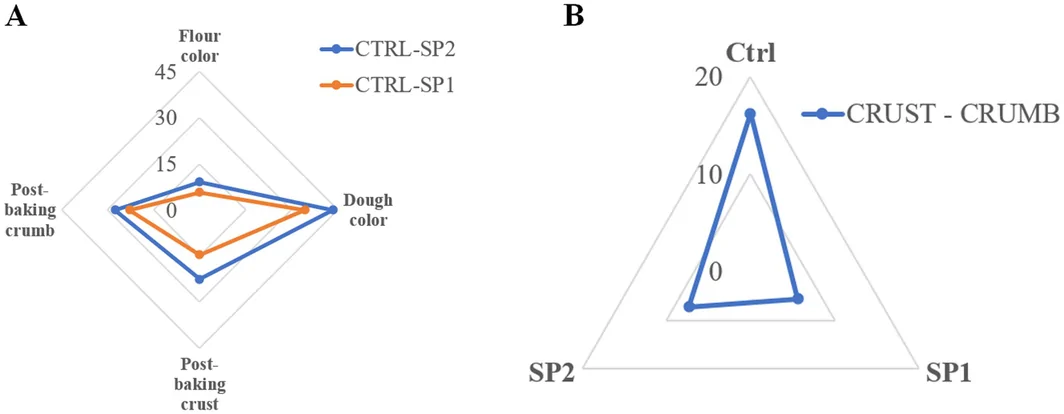
Radar graphs of (A) the color distance (*ΔE*) between the control samples and SF10 (1% w/w Spirulina) and SF20 (2% w/w Spirulina); of (B) the color distance (*ΔE*) between crust and crumb color.

Figure 2A shows the color distance (ΔE) between the control samples and SF10 and SF20 by means of a radar graph. Kneading leads to a distribution of the green‐blue pigment and a significant variation in color between the flours and the doughs. In addition, it is clearly noted that the ΔE values referred to the crust of the samples are lower than those referred to the crumb for both comparisons. Figure 2B shows comparisons between crust and crumb color within the same sample. The highest ΔE value was observed in the control sample (> 15), while the SF10 and SF20 samples showed values between 5.6 and 7.2, indicating a lower, though still perceptible, color variation to the human eye.

### 3.2 Results of TPA

Table 3 shows the results of the analysis carried out by the dynamometer on baked products (crumb). The textural properties of bread samples supplemented with Spirulina were compared to the control. In general, the addition of Spirulina did not significantly influence the texture parameters, except for the SF10 bread (1% w/w Spirulina), which showed significantly lower hardness (*p* < 0.05) compared to the control, thus indicating a softer texture. The SF20 sample (2% w/w Spirulina) had an intermediate value, not significantly different from either the control or SF10.

**TABLE 3:** Results of TPA on baked products. Control: Italian type “1” wheat flour (*W* = 300); SF10: Spirulina 1% w/w; SF20: Spirulina 2% w/w.

	Control	SF10	SF20	ANOVA (*p*)
Hardness (N)	13.95 ± 0.13 a	11.28 ± 2.19 b	13.00 ± 0.55 ab	0.02
Springiness	0.89 ± 0.01	0.85 ± 0.01	0.87 ± 0.02	n.s.
Cohesiveness	0.62 ± 0.01	0.64 ± 0.03	0.61 ± 0.01	n.s.
Gumminess (N)	8.64 ± 0.23	7.59 ± 1.23	7.85 ± 0.40	n.s.
Chewiness (N)	7.67 ± 0.17	6.48 ± 1.00	6.75 ± 0.33	n.s.

*Note:* Post hoc Tukey's test (*p* ≤ 0.05) was employed. Results are reported using different letters that label average values that are significantly different (a > b).

Abbreviation: n.s., not significant.

The lower hardness observed in SF10 bread aligns with the sensory results reported by Sanjari et al. (2018), who attributed this effect to the moisture‐retention capacity of algal fibers and their ability to delay starch retrogradation. However, it is worth noting that the literature does not show consistent results regarding this parameter.

Springiness, cohesiveness, gumminess, and chewiness did not show significant differences among the samples, indicating that Spirulina addition at 1% and 2% (w/w) did not substantially affect these parameters.

Spirulina incorporation did not markedly affect most of the TPA parameters measured under the tested conditions, in agreement with the findings of Hernández‐López et al. (2023). The 1% w/w enrichment level, however, resulted in significantly lower hardness than the control. Since specific volume was not determined, these results should not be interpreted as a complete assessment of bread structure or gas‐retention behavior.

### 3.3 Volatile Compounds

Table 4 shows the semi‐quantitative volatile profile of the baked samples, with compounds grouped into chemical classes, as determined by HS‐SPME‐GC‐EI‐MS. Since the results are expressed as relative percentages (sum = 100), differences in individual compounds should be interpreted with caution, as they may reflect both actual variation in abundance and redistribution within the overall volatile profile. Ethanol was excluded from the table because its abundance was several orders of magnitude higher than that of the other detected compounds.

**TABLE 4:** Volatile compounds (relative percentage to sum 100), extracted from baked products. Control: Italian type “1” wheat flour (*W* = 300); SF10: Spirulina 1% w/w; SF20: Spirulina 2% w/w.

	Aroma	Perception threshold	Control	SF10	SF20	One‐way ANOVA
**Alcohols**						
Isobutyl alcohol	Solvent, slightly fruity	High	1.63 a ± 0.10	0.59 b ± 0.03	0.48 b ± 0.02	0.001
Isoamyl alcohol	Banana, fusel, fruity	Intermediate	11.35 a ± 0.38	5.81 b ± 0.58	7.06 ab ± 2.15	0.048
3‐Ethoxy‐1‐propanol	Sweet, ethereal, alcoholic	High	0.85 a ± 0.15	0.50 ab ±0.04	0.34 b ± 0.02	0.022
1‐Octen‐3‐ol	Fungus, green, earthy	Very low	0.49 a ± 0.01	0.23 ab ±0.03	0.30 b ± 0.09	0.035
Benzyl alcohol	Flowery, almond, sweet	Low	0.17 ± 0.02	0.18 ± 0.03	0.16 ± 0.05	n.s.
Phenylethyl alcohol	Rose, honey, floral	Low	47.81 a ± 0.93	44.02 b ± 0.36	35.63 c ± 0.07	0.001
**Diols**						
2,3‐Butanediol (2R,3R)‐(−)	Sweet, creamy	Very high	11.21 b ± 1.02	21.36 a ± 1.02	17.29 ab ±1.81	0.015
2,3‐Butanediol (2R,3S)‐(+)	Sweet, slightly lactic	Very high	3.47 b ± 0.51	7.68 a ± 0.35	5.58 ab ±0.63	0.009
**Aldehydes**						
3‐Methylbutanal	Hazelnut, malt, toasted	Low	0.00 b ± 0.00	0.02 a ± 0.00	0.02 a ± 0.01	0.024
Nonanal	Fat, floral, citrusy	Very low	0.19 ± 0.07	0.18 ± 0.05	0.10 ± 0.02	n.s.
2,4‐Decadienal isomer	Herbaceous, green, rancid	Very low	0.13 a ± 0.03	0.02 b ± 0.01	0.03 b ± 0.02	0.023
(E,E) 2,4‐Decadienal	Fried, crust, hazelnut rancid	Very low	0.93 a ± 0.01	0.22 b ± 0.14	0.29 b ± 0.26	0.042
2‐Phenylethanal	Honey, rose, sweet	Low	1.47 b ± 0.25	1.51 b ± 0.10	2.91 a ± 0.04	0.004
**Ketones**						
Acetoin	Cream, butter	Intermediate	1.88 b ± 0.02	1.94 b ± 0.05	2.50 a ± 0.13	0.008
6,10,14‐Trimethylpentadecan‐2‐one	Soft, waxy, slightly floral	Low	0.02 ± 0.01	0.02 ± 0.01	0.02 ± 0.02	n.s.
**Acids**						
Acetic acid	Acidic, pungent	Low	1.86 ± 0.61	2.08 ± 1.70	1.48 ± 0.11	n.s.
**Esters and lactones**						
Ethyl hexanoate	Fruity, pineapple	Very low	0.09 a ± 0.01	0.02 b ± 0.00	0.03 b ± 0.00	0.001
Ethyl octanoate	Fruity, sweet, floral	Very low	0.69 a ± 0.05	0.22 b ± 0.01	0.23 b ± 0.09	0.007
Ethyl decanoate	Sweet, fruity, cherry	Low	0.41 a ± 0.00	0.13 b ± 0.00	0.18 b ± 0.02	4.63E−04
Ethyl dodecanoate	Fruity, creamy	Intermediate	0.11 a ± 0.04	0.01 b ± 0.00	0.02 ab ±0.01	0.039
Ethyl tetradecanoate	Sweet, waxy	High	0.02 ± 0.01	0.01 ± 0.01	0.02 ± 0.01	n.s.
γ‐Nonalactone	Coconut, cream, lactic acid	Low	0.86 ± 0.01	0.42 ± 0.18	0.62 ± 0.41	n.s.
**Hydrocarbons**						
Pentadecane	Oily, neutral	Very high	0.16 b ± 0.01	0.34 b ± 0.01	0.85 a ± 0.10	0.002
Hexadecane	Waxy, fatty	Very high	0.13 b ± 0.06	0.23 b ± 0.00	0.81 a ± 0.04	0.001
Heptadecane	Waxy, fatty	Very high	0.77 c ± 0.21	4.33 b ± 0.32	13.20 a ± 0.68	2.25E−04
8‐Heptadecene	Oily, algal, marine	Intermediate	0.08 b ± 0.01	0.12 b ± 0.01	0.34 a ± 0.02	0.001
**Pyrazines**						
Methylpyrazine	Toasted, hazelnut	Low	1.59 ± 1.11	0.94 ± 0.43	1.15 ± 0.32	n.s.
Ethylpyrazine	Toasted, cereal	Low	1.09 ± 0.43	0.63 ± 0.12	0.62 ± 0.09	n.s.
2‐Ethyl‐6‐methylpyrazine	Toasted, earthy	Low	0.25 ± 0.20	0.18 ± 0.07	0.30 ± 0.14	n.s.
2‐Ethyl‐5‐methylpyrazine	Popcorn, toasted	Very low	0.17 ± 0.12	0.14 ± 0.06	0.22 ± 0.14	n.s.
2‐Ethyl‐3‐methylpyrazine	Toasted, vegetable	Very low	0.18 ± 0.15	0.08 ± 0.02	0.15 ± 0.11	n.s.
Trimethylpyrazine	Coffee, cereal, cocoa	Very low	0.11 ± 0.05	0.11 ± 0.03	0.16 ± 0.08	n.s.
2,6‐Diethylpyrazine	Bread, cereal	Very low	0.06 ± 0.04	0.05 ± 0.03	0.10 ± 0.05	n.s.
2‐Ethyl‐3,5‐dimethylpyrazine	Coffee, toast	Very low	0.05 ± 0.06	0.05 ± 0.03	0.11 ± 0.07	n.s.
**Furanic and pyranic compounds**						
2‐Pentylfuran	Green, cucumber, vegetable	Low	1.31 a ± 0.04	0.68 b ± 0.02	0.61 b ± 0.08	0.002
Furfural	Almond, bread, caramel	Very low	3.83 a ± 0.19	0.99 b ± 0.68	0.65 b ± 0.09	0.013
Furfuryl alcohol	Cake, bread, yeast	Low	1.44 a ± 0.12	0.82 b ± 0.12	0.71 b ± 0.05	0.010
Maltol	Caramel, cotton candy	Very low	0.50 b ± 0.03	0.49 b ± 0.05	0.81 a ± 0.03	0.006
Hydroxymethylfurfural	Sweet, cooked sugar	Intermediate	0.02 ± 0.00	0.02 ± 0.01	0.01 ± 0.00	n.s.
**Apocarotenoids and C**_**13**_ **‐norisoprenoids**						
β‐Cyclocitral	Lemongrass, citrus fruits	Low	0.00 c ± 0.00	0.03 b ± 0.00	0.07 a ± 0.00	3.69E−04
Safranal	Spicy, saffron	Very low	0.01 c ± 0.00	0.03 b ± 0.00	0.06 a ± 0.00	1.37E−04
α‐Ionone	Violet, floral	Low	0.00 b ± 0.00	0.02 a ± 0.00	0.01 a ± 0.00	0.010
β‐Ionone	Violet, floral, sweet	Very low	0.04 b ± 0.00	0.25 ab ±0.04	0.72 a ± 0.22	0.028
5,6‐Epoxide‐β‐ionone—	Floral, complex	Intermediate	0.02 b ± 0.01	0.10 b ± 0.01	0.28 a ± 0.03	0.002
3,4‐Dehydro‐β‐ionone	Violet, citrus	Low	0.00 c ± 0.00	0.01 b ± 0.00	0.02 a ± 0.00	3.53E−04
Dihydroactinidiolide	Black tea, tobacco, sweet	Low	0.01 c ± 0.01	0.06 b ± 0.01	0.17 a ± 0.02	0.002
**Pyrroles**						
2‐Acetylpyrrole	Bread, hazelnut, toasted	Low	0.21 ± 0.07	0.20 ± 0.06	0.37 ± 0.02	n.s.
**Phenols**						
Phenol	Pungent, smoky, medicinal notes	Very low	0.51 a ± 0.03	0.28 b ± 0.04	0.26 b ± 0.07	0.024
2‐Methoxy‐4‐vinylphenol	Spicy, smoky, cloves	Low	2.04 ± 0.24	0.97 ± 0.30	1.45 ± 0.21	n.s.

*Note:* Perception threshold (μg/kg): very low < 1; low 1–10; intermediate 10–100; high 100–1000; very high > 1000. Post hoc Tukey's test (*p*‐value ≤ 0.05) was employed. Results are reported using different letters that label average values that are significantly different (a > b > c).

Abbreviation: n.s.: not significant.

Several alcohols commonly associated with fermentation were generally more abundant in control breads than in Spirulina‐enriched samples. Since these compounds were largely absent in Spirulina powder, their occurrence in bread can be mainly attributed to fermentation‐related processes. This pattern suggests that Spirulina addition influenced the formation of fermentation‐related volatile compounds.

Conversely, the two 2,3‐butanediol isomers tended to increase in Spirulina‐enriched breads, and acetoin was significantly higher in SF20, whereas ethyl esters were relatively less abundant. Overall, this pattern suggests that Spirulina addition influenced the formation of fermentation‐related volatile compounds (Bao et al. 2018; Ji et al. 2011). However, because the data were expressed as semi‐quantitative relative percentages and no direct measurements of yeast metabolism, fermentation kinetics, CO_2_ evolution, ethanol production, or microbial viability were performed, these observations should be interpreted cautiously and do not demonstrate a specific metabolic rerouting. Further studies integrating volatile, microbiological, and fermentation measurements will be necessary to clarify the mechanisms underlying these differences.

Among aldehydes, 3‐methylbutanal was detected only in Spirulina‐enriched samples, although at very low relative abundance. This finding may be consistent with the higher leucine availability previously reported in Spirulina‐enriched bread systems (Montevecchi et al. 2022). (E)‐2‐Nonenal and the two 2,4‐decadienal isomers, commonly associated with lipid oxidation and fatty notes in bread, were significantly more abundant in the control samples. This pattern may be consistent with a possible mitigating effect of Spirulina on oxidative processes (Mohammadi et al. 2022). 2‐Phenylethanal was significantly higher in SF20. As this compound may derive from phenylalanine degradation, its increase could be consistent with the amino acid composition of Spirulina. However, its actual sensory contribution in the present breads should be interpreted cautiously.

Regarding organic acids, no statistically significant differences in acetic acid levels were observed between samples. This is expected, as acetic acid primarily derives from the microbial metabolism of sugars, and Spirulina contributed a negligible amount of carbohydrates.

Pyrazines, which are typical Maillard‐derived compounds associated with toasted and roasted notes, did not differ significantly among samples. Another polycyclic aromatic nitrogen heterocycle, 2‐Acetylpyrrole, was more abundant in Spirulina‐containing samples and may be associated with the presence of Spirulina‐derived precursors or pigments.

Among furanic compounds, furfural and furfuryl alcohol were more abundant in the control samples. This pattern may be related to differences in pH and thermal reaction pathways during baking. 2‐Pentylfuran, a compound associated with green and vegetal notes, was present at low relative abundance and was higher in the control samples. This difference may be related to the antioxidant properties of Spirulina.

Maltol was significantly higher in SF20. As maltol is a heat‐derived compound associated with sweet, caramel‐like notes, this result suggests that Spirulina addition also affected some thermal reaction products.

Some volatile substances can be considered as “varietal,” deriving from the presence of Spirulina. These include, in particular, some alkanes, alkenes, and carotenoid derivatives. Several hydrocarbons, particularly pentadecane, hexadecane, heptadecane, and 8‐heptadecene, were more abundant in Spirulina‐enriched breads, especially in SF20. These compounds may be considered markers of Spirulina addition, although their direct sensory contribution is likely limited because of their high perception thresholds.

Numerous carotenoid‐derived compounds were found in Spirulina powder as well as in experimental samples, always in significantly higher relative amounts in samples containing the highest amount of Spirulina. β‐Cyclocytral represents the cyclic form of citral (a mixture of two isomers, neral and geranial) which gives the typical citrus note, as well as the main component of lemongrass essential oil. Several in vitro experiments have shown that the oxidation of β‐carotene through O_2_ produces various volatile compounds such as β‐cyclocytral, β‐ionone, dihydroactinidiolide, and α‐ionene (Ramel et al. 2012).

Safranal was not detected in Spirulina powder, while it was found in both Spirulina‐enriched samples. The presence of this molecule, the main responsible for the saffron flavor, can be linked to the baking process due to the degradation that occurs in carotenoid pigments, in particular zeaxanthin. During the drying process, by thermal or enzymatic action, zeaxanthin produces picrocrocin and finally safranal (Carmona et al. 2006).

Ionones belong to a class of chemical compounds, known as C_13_‐norisoprenoids, also derivatives of carotenoids, present in various essential oils characterized by floral notes, violet‐like scents with a woody note for the β isomer (Francezon et al. 2021; Jia et al. 2024; Zhao et al. 2024).

Dihydroactinidiolide is a lactone derived from the second oxidation of β‐ionone through the intermediate β‐ionone‐5,6‐epoxide (Havaux 2014). It is a volatile terpene that has a sweet, tea‐like odor and has been identified as one of the volatile components of selected cyanobacteria strains (Stevens and Merrill 1981).

Compounds such as β‐ionone, β‐cyclocitral, dihydroactinidiolide, and safranal may potentially be perceived as off‐flavors in baked products when present at high concentrations, despite contributing positively to the overall aroma profile at trace levels. Specifically, β‐ionone can impart overly intense floral or violet‐like notes, β‐cyclocitral may introduce pungent green or earthy aromas, dihydroactinidiolide is associated with tobacco‐like or woody nuances, and safranal can result in sharp, medicinal, or excessively spicy impressions that may disrupt the desired sensory balance of bakery items.

Among phenolic compounds, phenol was significantly more abundant in the control samples. This difference may be associated with the different composition of the Spirulina‐enriched matrices, although the mechanisms underlying this effect were not specifically investigated in the present study.

### 3.4 Consumer Attitudes Toward Algae‐Enriched Bakery Products

The survey explored general attitudes and purchase intentions regarding algae‐enriched bakery products and should not be interpreted as a direct measure of liking for the specific bread samples developed in the present study.

The sample mainly comprised young adults (18–34 years, 46.7%), women (60.9%), and university graduates (59.6%), with students (38.7%) and office workers (22.7%) as the most represented occupational groups. Almost all participants (98.2%) were regular consumers of baked goods, with consumption reported as daily (27.6%), two to three times per week (44.0%), or less than once per week (27.6%).

Half of the respondents were unfamiliar with algae‐enriched bakery products, and only 3% reported occasional consumption. Positive drivers of acceptance included nutritional profile, crispness, and environmental sustainability, whereas marine‐like flavor, fishy odor, and bitterness were identified as main barriers, consistent with previous reports (Bleakley and Hayes 2017; Birch et al. 2019; Rabitti et al. 2024). Despite these concerns, 92.8% of participants expressed willingness to try and purchase such products, motivated by curiosity, perceived health benefits, and sustainability (Graça et al. 2019; Lafarga et al. 2021).

Overall, these results indicate a favorable consumer openness toward algae‐enriched bakery products, provided sensory challenges are addressed. This context supports the *rationale* of the present work, where the sensory evaluation of Spirulina‐enriched bread is used to directly explore the barriers highlighted in the survey.

### 3.5 Sensory Analysis

**FIGURE 3:**
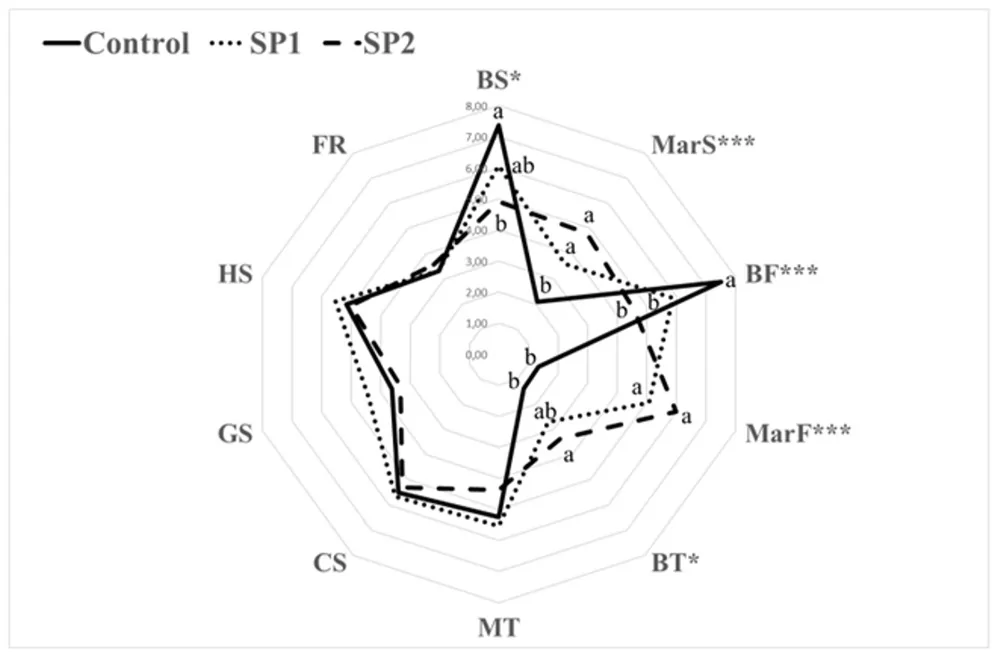
Sensory profile of the products: Control bread obtained with Italian type “1” wheat flour (*W* = 300); SF10 bread obtained by adding *Spirulina* 1% w/w; SF20 bread obtained by adding *Spirulina* 2% w/w. BF, Bread flavor; BS, Bread smell; BT, Bitterness; CS, Cohesiveness; FR, Fibrousness; GS, Gumminess; HS, Hardness; MarF, Marine flavor; MarS, Marine smell; MT, Moist.

Figure 3 shows the results of the sensory analysis performed on the sample set.

Because each judge evaluated each sample only once, judge repeatability and the judge × sample interaction could not be estimated. Therefore, additional analyses compatible with the available design were performed to assess judge‐related variability and agreement among assessors. For each sensory attribute, an additive model including sample and judge effects was applied. The model showed a significant judge effect for BS, BT, HS, CS, and FR, indicating differences in scale use among assessors. In addition, Kendall's coefficient of concordance (*W*) showed significant agreement among judges for the main attributes affected by formulation, namely BS (*W* = 0.391, *p* = 0.014), BF (*W* = 0.669, *p* = 0.001), MarS (*W* = 0.415, *p* = 0.010), MarF (*W* = 0.615, *p* = 0.001), and BT (*W* = 0.352, *p* = 0.021) (Table S1).

Significant differences were observed for smell, flavor, and taste attributes (*p* ≤ 0.05). In particular, the Spirulina‐enriched breads were perceived as significantly more bitter (BT) than the control. Spirulina has been reported to exhibit off‐flavors, such as fishy and mushroom‐like notes, which are often associated with greater bitterness and umami intensity (Rabitti et al. 2024). Fermentation with lactic acid bacteria has been shown to reduce undesirable bitter peptides, thereby mitigating these bitter perceptions (Chen and Yang 2021).

Consistently, marine notes (MarS and MarF) were mainly detected in the breads containing Spirulina. These perceptions may have partially masked the typical smell and flavor of bread because of the presence of algae. In contrast, no significant differences were observed between the Spirulina‐enriched samples and the control for the texture‐related sensory attributes CS, GS, HS, and FR. This suggests that Spirulina addition did not markedly affect the perceived texture of the breads under the conditions of the present study, unlike what was reported by Sanjari et al. (2018).

Although the sensory evaluation was carried out under green lighting to reduce visual bias, some panelists still described the crust of Spirulina‐enriched bread samples as differing in appearance from the control. In particular, SF10 and SF20 samples were more frequently associated with lighter and darker, respectively. However, these observations should be interpreted cautiously since the use of green lighting was intended to mask color differences rather than to provide an ecologically valid assessment of visual attributes under normal viewing conditions.

In general, the judges found no differences in smell, flavor, and taste between the two samples enriched with Spirulina, suggesting that, within the tested range, the two Spirulina levels produced comparable sensory effects for these attributes. However, Montevecchi et al. (2022) previously reported that Spirulina concentrations above 2% w/w caused substantial dough‐handling problems because of the chemical characteristics of Spirulina and the high dough hydration, resulting in excessive stickiness. Therefore, lower‐hydration formulations and drier baked products, such as breadsticks, could be considered in future studies to explore higher Spirulina levels without markedly impairing dough workability. Even so, appearance, dark color, and olfactory and taste attributes would remain critical aspects to be addressed. In addition, the descriptive sensory analysis identified bitterness and marine notes in Spirulina‐enriched breads, which may represent barriers to acceptance; however, consumer liking was not directly assessed in the present study.

## 4 Conclusions

This study extends previous research on Spirulina‐enriched wheat systems by providing an integrated assessment of color, instrumental texture, volatile composition, and descriptive sensory profile in bread. Spirulina incorporation markedly modified the chromatic properties of the samples: the dough acquired an intense green coloration due to cyanobacterial pigments, whereas baking induced substantial color changes, likely involving pigment degradation and the development of brown tones in both crust and crumb.

Spirulina addition also modified the volatile profile of bread, including an increase in some carotenoid‐derived compounds such as β‐ionone, β‐cyclocitral, dihydroactinidiolide, and safranal. However, because the volatile data were expressed as relative percentages, these differences should be interpreted as semi‐quantitative compositional changes rather than absolute concentration differences. Moreover, no direct measurements of fermentation kinetics or yeast metabolism were performed.

Most instrumental texture parameters did not differ significantly among samples; however, bread containing 1% w/w Spirulina showed significantly lower hardness than the control. Since specific volume was not determined, the present results should not be regarded as a complete assessment of bread structure or gas‐retention behavior.

The descriptive sensory analysis revealed increased bitterness and marine notes in Spirulina‐enriched breads, indicating that these attributes may represent important barriers to acceptance. Although the survey suggested general consumer openness toward algae‐enriched bakery products, it assessed attitudinal perceptions rather than liking of the specific breads developed in this study.

The present work provides an initial characterization of several quality changes associated with low‐level Spirulina incorporation in bread, but it does not provide direct nutritional characterization of the final baked products and does not include mitigation strategies for sensory drawbacks. Further studies are needed to optimize formulations, verify the nutritional characteristics of the final breads, and assess consumer acceptance directly.

## Author Contributions

**Francesca Masino:** formal analysis, investigation, funding acquisition, writing – original draft, writing – review and editing, methodology. **Alessandra Riggio:** formal analysis, investigation, writing – original draft. **Marco Gammaitoni:** formal analysis, investigation, writing – original draft. **Giulia Santunione:** validation, writing – review and editing. **Fabio Licciardello:** methodology, formal analysis. **Emanuela Lo Faro:** methodology, formal analysis. **Andrea Antonelli:** validation, funding acquisition, writing – review and editing, resources. **Giuseppe Montevecchi:** conceptualization, methodology, investigation, writing – review and editing.

## Funding

This work was partially funded by PR‐FESR Emilia‐Romagna 2021–2027, Asse 1, Azione 1.1.2 “Supporto a progetti di ricerca collaborativa dei laboratori di ricerca e delle università con le imprese.” Bando Laboratori 2023–2024 per progetti di ricerca industriale strategica rivolti agli ambiti prioritari della SS3 2023–2024. Project name: ALGENFOR—Uso di alghe per una nuova generazione di prodotti da forno [CUP E87G22000630003], https://www.algenfor.eu/.

## Ethics Statement

The study was approved by the Ethics Committee “Comitato etico per la ricerca dell'Università di Modena e Reggio Emilia (CEAR)” (approval date: 09/01/2025; Protocol No. 4569). All participants provided informed consent prior to participation. All members in the sensory analysis participated voluntarily after signing an informed consent form, which included a description of the study and any potential risks. Furthermore, all data were kept confidential and handled in accordance with privacy regulations.

## Conflicts of Interest

The authors declare no conflicts of interest.

## Supporting information


**Table S1:** Additive model and Kendall's coefficient of concordance (*W*) for sensory attributes.

## Data Availability

Data will be made available on request.
