# Supplementary material for: Effects of Spirulina Enrichment on Bread Quality: Color, Texture, Volatile, and Sensory Properties
Source: Food Sci Nutr. 2026 Sep 15;14(9):e72345. doi: 10.1002/fsn3.72345 (PMC13575868; doi:10.1002/fsn3.72345)
Supplement: Supplementary file 1 [file FSN3-14-e72345-s001.docx]

**Supplementary Table S1. Additive model and Kendall’s coefficient of concordance (W) for sensory attributes.**

| **Attribute** | **Sample effect**  **(*p-value*)** | **Judge effect**  **(*p-value*)** | **Kendall**  **W** | **Kendall**  ***p-value*** |
| --- | --- | --- | --- | --- |
| *BS* | 0.031 | < 0.001 | 0.391 | 0.014 |
| *BF* | < 0.001 | 0.058 | 0.669 | 0.001 |
| *MarS* | < 0.001 | 0.190 | 0.415 | 0.010 |
| *MarF* | < 0.001 | 0.208 | 0.615 | 0.001 |
| *BT* | 0.004 | 0.005 | 0.352 | 0.021 |
| *MT* | 0.222 | 0.149 | 0.104 | 0.319 |
| *HS* | 0.579 | 0.006 | 0.040 | 0.643 |
| *GS* | 0.107 | 0.098 | 0.078 | 0.424 |
| *CS* | 0.711 | < 0.001 | 0.016 | 0.843 |
| *FR* | 0.523 | < 0.001 | 0.035 | 0.683 |
